# Progress in Ameliorating Beneficial Characteristics of Microbial Cellulases by Genetic Engineering Approaches for Cellulose Saccharification

**DOI:** 10.3389/fmicb.2020.01387

**Published:** 2020-06-24

**Authors:** Anica Dadwal, Shilpa Sharma, Tulasi Satyanarayana

**Affiliations:** Department of Biological Sciences and Engineering, Netaji Subhas University of Technology, New Delhi, India

**Keywords:** genetic engineering, protein engineering, cellulose saccharification, cellulases, thermostability, multifunctional cellulases, cellulosomes

## Abstract

Lignocellulosic biomass is a renewable and sustainable energy source. Cellulases are the enzymes that cleave β-1, 4-glycosidic linkages in cellulose to liberate sugars that can be fermented to ethanol, butanol, and other products. Low enzyme activity and yield, and thermostability are, however, some of the limitations posing hurdles in saccharification of lignocellulosic residues. Recent advancements in synthetic and systems biology have generated immense interest in metabolic and genetic engineering that has led to the development of sustainable technology for saccharification of lignocellulosics in the last couple of decades. There have been several attempts in applying genetic engineering in the production of a repertoire of cellulases at a low cost with a high biomass saccharification. A diverse range of cellulases are produced by different microbes, some of which are being engineered to evolve robust cellulases. This review summarizes various successful genetic engineering strategies employed for improving cellulase kinetics and cellulolytic efficiency.

## Introduction

**FIGURE 1:**
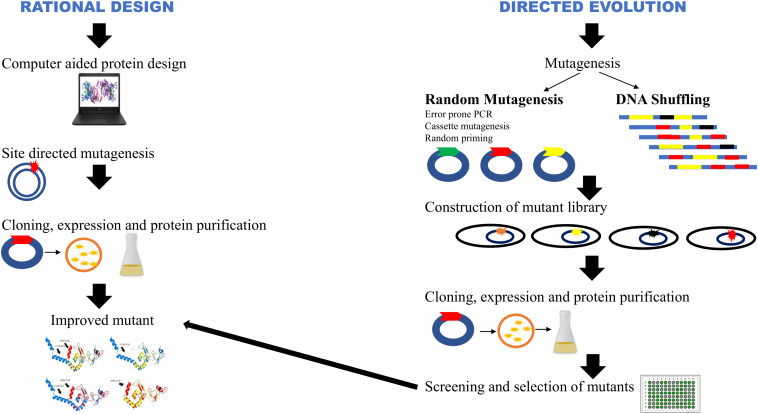
Rational design and directed evolution strategies for genetic engineering. (Adopted with permission from Bornscheuer and Pohl, 2001).

**FIGURE 2:**
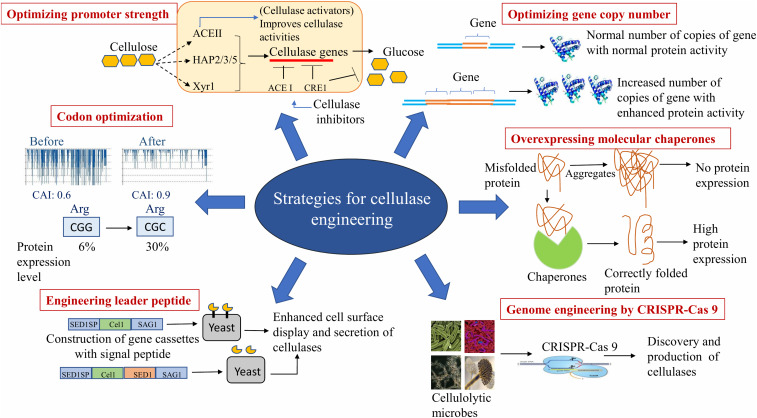
Strategies employed in cellulase engineering.

Lignocellulosic biomass, is an abundantly available renewable energy resource. Hectic efforts are being made to develop technologies to produce bioethanol, biobutanol, and other products from crop residues. Due to highly ordered compact structure of cellulose microfibrils of lignocellulosics, there is a need to loosen the matrix in order to make crystalline cellulose accessible for degrading enzymes (Arantes and Saddler, 2010). Physico-chemical pretreatment of biomass is required in order to improve saccharification. Despite several methods described in the literature for the pretreatment of lignocellulosics, there is no single universal method that can be used for all types of lignocellulosic biomass because of an immense variation in their composition and structure (Capolupo and Faraco, 2016). Due to recalcitrant nature of lignocellulosics, the enzyme quantity required to hydrolyze biomass is several-fold higher than that for starch (Balan, 2014). Using process engineering approaches, commercial enzyme companies have made impressive growth in producing new generation enzymes in the recent years. Low enzymatic activity and high cost of cellulases are the bottlenecks for industrial use of cellulases (Sukumaran et al., 2005). It is understood that the cost of 2nd generation (2G) bioethanol can only be reduced by bringing down the cost component of cellulases (Klein-Marcuschamer et al., 2012). The production of cellulases at low cost with improved efficiency is, therefore, an immediate requirement. There have been several attempts in applying genetic engineering in the production of a repertoire of cellulases at low cost with high biomass saccharification efficiency; some of these processes are quite laborious (Singh et al., 2017). Despite the voluminous work done till date, there are a number of problems that need to be addressed. The resurgence in the availability of whole genome sequences of several important cellulase-producing microbial strains and information on their genetic functions have paved the way for targeted modifications (Singh et al., 2017). Figure 1 depicts two strategies: rational design and directed evolution, which are widely used for improving the characteristics of individual cellulase components. Rational design approach for protein engineering via computational methods is facilitating developments in this field. Modifications at various levels such as at the gene sequences (site directed mutagenesis), promoters, transcriptional factors, gene copy number, codons, chaperones and leader peptide, and at structural levels such as in glycosylation and enzyme folding have led to the evolution of robust cellulase-producing strains (Pei et al., 2012; Zou et al., 2012; Amore et al., 2017; Li et al., 2017, 2018) (Figure 2).

**FIGURE 3:**
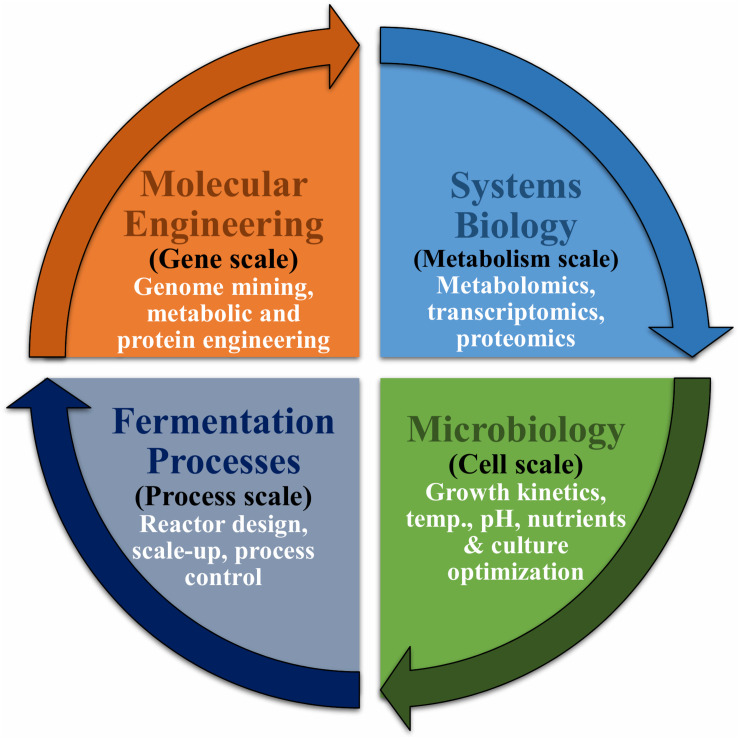
Engineering strategies in scale-up of production of bioethanol and other products.

Considering the various economic and desirable qualities of cellulases in cellulose saccharification efficiency such as high thermostability, resistance to end product inhibition, high specific activity and catalytic efficiency a lot of investigations had been carried out over the years to develop efficient enzymes utilizing various genetic/metabolic engineering techniques (Cao et al., 2018; Han et al., 2018; Saavedra et al., 2018; Zheng et al., 2018; Bashirova et al., 2019; Yoav et al., 2019; Adebami and Adebayo-Tayo, 2020; Li et al., 2020b; Wu et al., 2020). Significant efforts have also been made in discovery of novel cellulases using metagenomics (Garg et al., 2016; Song et al., 2016; Zhao et al., 2017). The development of consolidated bioprocessing (CBP) technology for converting cellulosic biomass directly into bio-products is of high interest as the process that does not require the addition of cellulolytic enzymes (Liu et al., 2018; Xin et al., 2019). Recent investigations are thus focused on engineering CBP microbes that can utilize cellulosic biomass directly to produce bioproducts such as bioethanol (Chung et al., 2015; Yang X. et al., 2015; Guo et al., 2017; Li et al., 2020a). Utilization of multifunctional enzymes is another acclaimed approach. The multifunctional enzymes, are a single gene product possessing several glycoside hydrolase activities. It is considered to reduce the costs of enzyme production, purification as well as enzyme loading compared to that with the cooperative action of several individual glycoside hydrolases (Teeravivattanakit et al., 2016). Development and engineering of multifunctional enzymes and cellulosomes are crucial for developing next generation enzymes for biomass deconstruction (Brunecky et al., 2020). In addition to this, the conventional strategies that are employed in scaling up of the production of bioethanol and other products are depicted in Figure 3. This review, therefore, focuses on various genetic and metabolic engineering approaches, which have been used for improving cellulase production and characteristics. Although genetic modification is a potent technique for evolving catalytically efficient and high cellulase-producing microbial strains, novel cellulases can also be generated through metagenomics (Xia et al., 2013; Yang C. et al., 2016).

## Cellulose-Degrading Enzymes

Lignocellulosic plant biomass comprises mainly of three types of polymers: cellulose, hemicellulose and lignin. Cellulose is a linear polysaccharide made of β-1, 4-linked glucose units. The cellulose chains are packed into microfibrils via hydrogen bonding and van der Waals interactions (Chundawat et al., 2011). These microfibrils are crystalline and insoluble in nature, thus requiring enzymatic digestion. Complete depolymerization of cellulose yields glucose. The recalcitrance of cellulose to enzymatic hydrolysis is a limitation (Chundawat et al., 2011). Based on the substrate specificity and catalytic domains, cellulases are classified into three major classes: β-1,4-endoglucanases (EC 3.2.1.4), exoglucanases or cellobiohydrolases [acting on non-reducing end (EC 3.2.1.91), reducing-end (EC 3.2.1.174)] and β-glucosidases (EC 3.2.1.21) (Sharma et al., 2016). The cellulose degradation mechanisms involve the synergistic action of three cellulases. Endo-1,4-β-glucanases (egls) randomly hydrolyzes internal bonds in cellulose chain to generate new reducing and non-reducing ends. Exo-1,4-β-glucanases/cellobiohydrolases (cbhs) attack the reducing or non-reducing ends of the cellulose polymer to liberate cellobiose. β-Glucosidases (bgls) hydrolyze cellobiose, the major product of the exoglucanase, to glucose (Horn et al., 2012). Cellulases belong to the glycoside hydrolase (GH) family. The genes encoding glycohydrolases (GHs) are classified in CAZy (Carbohydrate Active enZymes) database, which are primary enzymes that cleave glycosidic bonds by adding water molecules (Horn et al., 2012).

Cellulases are a part of a large group of GHs categorized into several families based on their amino acid sequences, 3D structures, and catalytic mechanisms (Park et al., 2017). Carbohydrate-Active EnZymes (CAZy^1^) database contains GH families in which the cellulases are included. The egls are classified into GH5, GH6, GH7, GH9, GH12, GH44, GH45, GH48, GH51, GH74, and GH124; cbhs in GH6, GH7, GH9, and GH48, while bgls are classified mainly in GH1, GH3, GH9, and GH30 (see footnote 1). Glycoside hydrolases present in some bacterial and fungal species exhibit multi-modular structure which consists of a carbohydrate binding module (CBM), a linker and a catalytic domain (Payne et al., 2015).

Chitin Binding Protein 21 (CBP21) that acts on crystalline chitin occurring in the shells of insects and crustaceans, was found to increase substrate accessibility and potentiates hydrolytic enzymes (Horn et al., 2012). CBP21 protein belongs to family 33 carbohydrate-binding module (CBM33) in the CAZy database. Genes encoding CBM33 are generally found in bacteria and viruses but are rarely present in eukaryotes. Fungi are known to produce proteins structurally similar to CBM33, which are currently classified as family 61 glycoside hydrolases (GH61) (Horn et al., 2012). The GH61 includes copper dependent Lytic Polysaccharide Monooxygenases (LPMOs), which are known to act synergistically with cellulases (Hemsworth et al., 2015). LPMOs are present in both bacteria and fungi. They are originally reported to be hydrolases, thus were annotated under GH61 family. Recently, they have been classified as auxiliary activity family 9 (AA9, formerly GH61), family 10 (AA10, formerly CBM33) and family 11 (AA11) (Morgenstern et al., 2014). These enzymes oxidatively cleave the C1 and/or C4 position of glucose units in the chain through a flat active site with a centrally located copper atom. The product is oxicellulose with a normal non-reducing end and a C1-oxidized end or native reducing end. As LPMOs oxidize lignocellulosic substrates without the addition of an external electron donor, lignin has been speculated to be the electron supplier for the activity of LPMOs. Currently, commercial cellulase preparations include LPMOs because their presence significantly reduces enzyme loading (Müller et al., 2018).

An oxidative system has been discovered, where extracellular flavocytochromes [cellobiose dehydrogenases (CDHs)] cooperate with LPMOs to catalyze redox-mediated glycosidic bond cleavage in crystalline cellulose and hemicelluloses (Langston et al., 2011). The CDH-LPMO system enhances the cellulose degradation efficiency; the mechanism of this has not yet been understood. CDHs comprise a haem β-binding cytochrome domain (CYT) which is linked by flavin adenine dinucleotide (FAD)-binding dehydrogenase domain (DH) with the help of long, flexible linker. Class-I CDHs are produced by basidiomycetes which lack additional domains, whereas class-II CDHs are produced by ascomycetes which may or may not possess Carbohydrate Binding Domain (CBM), corresponding to classes IIA and IIB, respectively (Meier et al., 2018).

Non-catalytic proteins known as expansins consist of two domains D1 and D2, which are connected by peptide linker. D1 domains shows structural and sequence similarity with the catalytic site of GH45 (the GH family to which egls belongs). In contrast, D2 domain display the presence of conserved aromatic and polar residues present in CBM (Yennawar et al., 2006). Expansins lacks hydrolytic activity and thought to act like a zipper opening the cross-linking of cellulose microfibrils by loosening the tightly bound chains, which in turn enhance cellulose accessibility to cellulases (Arantes and Saddler, 2010). Expansin like proteins have been reported in bacteria and fungi. The addition of expansin along with *Trichoderma reesei* cellulases led to 13% enhancement in cellulose conversion of the pretreated yellow poplar sawdust as compared to the sugar yield achieved with cellulase only (Baker et al., 2000). Kerff et al. (2008) reported a protein (EXLX1) secreted by *Bacillus subtilis*, which is a member of the expansin super family based on its structural similarity to plant expansins. The combination of recombinant EXLX1 protein and commercial *T. reesei* cellulases led to enhanced cellulolytic activity while hydrolyzing filter paper (Arantes and Saddler, 2010). Another example of expansin-like proteins is swollenin isolated from *T. reesei*. The protein plays a similar role like expansins in swelling the cellulose fibrils (Arantes and Saddler, 2010). Andberg et al. (2015) reported swollenin to exhibit hydrolytic activity against cellulosic substrates similar to both endoglucanases and cellobiohydrolases with cellobiose as the major degradation product liberated from β-glucan and cello-oligosaccharides.

The complete enzymatic degradation of lignocelluloses is achieved by concerted action of ligninases (manganese peroxidase, versatile peroxidase, lignin peroxidase and laccase), cellulases (endoglucanase, exoglucanases and β-glucosidase) and various hemicellulases. Polysaccharide esterases remove methyl, acetyl and phenolic esters present in lignocellulosic biomass, thus facilitating GHs to act on cellulose and hemicelluloses (Cragg et al., 2015). In some cases, polysaccharide lyases are also known to depolymerize polysaccharides (Villay et al., 2012).

In the past few years, several studies have emphasized on the isolation and development of fungal enzyme mixtures, which can be custom-made to efficiently hydrolyze recalcitrant lignocellulose into sugars. Enzymatic degradation alone is not sufficient to achieve higher sugar yields. Due to highly ordered regions of cellulose microfibrils, there is a need to loosen the matrix in order to enhance the accessibility of crystalline structure to cellulose-degrading enzymes (Brodeur et al., 2011). The pretreatment of biomass is therefore required, although no single pretreatment technology can be successfully applied for all types of biomass. The investigations on the pretreatment aspects are also required for attaining a high cellulose saccharification.

## Factors Governing Thermostability and Kinetics of Cellulases

In order to design stabilized and robust biocatalysts, the enzymes from thermophilic microbes (thermozymes) have been extensively studied for understanding the molecular basis of thermal adaptation. The biochemical and structural analyses have revealed that thermostability is imparted by the concerted action of different structural features rather than a single unique feature (Cobucci-Ponzano et al., 2010). Various stabilization factors such as a lower number of loops and cavities, increased number of ion pairs, large number of solvent molecules buried in cavities of the protein core, a higher number of proline residues in loops, a reduced ratio of protein surface area to protein volume, greater degree of oligomerization and an increased hydrophobic interactions have been predicted for the enhanced thermostability of thermozymes (González-Siso, 2019). The composition of amino acids is also a determining factor in thermostability of enzymes. A decrease in cysteine and an increase in the number of acidic residues, mainly glutamic acid, are thought to play a role in enhanced thermostability of enzymes (Voorhorst et al., 1995). It is also known that generally the stability of a protein increases with the presence of helix-forming amino acids into the α-helices and decreases with the presence of helix-breaking amino acids (Singh and Hayashi, 1995). Rawat et al. (2015) reported hydrophobic and aromatic residues are critical in protein thermal stability in the egl (GH12) of *Aspergillus niger*.

The disulfide bonds are also known to affect the thermostability of the enzymes (Kim and Ishikawa, 2013). Yang H. et al. (2018) observed that the disulfide bond at C12-C48 was critical for thermal adaptation and refolding of a novel thermostable GH45 cellulase (TaCel45) of thermophilic fungus *Thielavia arenaria* XZ7. The mutant displayed decreased optimal temperature and Tm values of 50 and 60.2°C; these were 90–100 and 68°C in the wild-type strain (Yang H. et al., 2018). Charged residues present in the enzyme structure contribute to structural integrity and in turn to thermostability. In another investigation, the introduction of additional disulfide bridges in the catalytic module of *Talaromyces emersonii* Cel7A resulted in mutants (G4C/A70C, N54C/P191C and T243C/A375C) with improved thermostability (Voutilainen et al., 2009). For the glucose tolerance of β-glucosidase, the structure between subsites +1 and +2 is critical (Matsuzawa et al., 2016; Goswami et al., 2017).

Recently, tunnel engineering has been attempted as a strategy to improve the catalytic properties of the enzymes with buried active sites (Lu L. et al., 2019). The tunnel like structure was reshaped through multi-site saturated mutagenesis that led to increase in specific activity (80–340%) of xylanases from *Bacillus* sp. This strategy appears to be beneficial in enhancing biomass degrading abilities of cellulases with buried active sites.

Understanding the structural features responsible for thermostability in cellulases is complicated because of a range of structural and sequence diversity in these enzymes. Extensive knowledge on thermostability would help in engineering enzymes with enhanced activity.

## Genetic Engineering Approaches for Improving Cellulase Kinetics and Activities

Cellulases are gaining immense attention not only in biofuel industry but also in other industrial processes such as paper manufacturing, fruit juice extraction, as detergent enzymes, in pharmaceuticals and animal feed additives (Dadwal et al., 2019). With such wider applications (Table 1), cellulases are likely to become one of the largest groups of industrially useful enzymes worldwide. For the utilization of cellulases on an industrial scale, some obstacles need elimination. One of the major limitations of cellulases is their high cost, which accounts for more than 20% of the total ethanol production cost from lignocellulosics according to the evaluation by the United States National Renewable Energy Laboratory (NREL) (Bušić et al., 2018). Low enzyme catalytic efficiency and thermostability are the other drawbacks. Moreover, a mix of different enzymes is required for efficient biomass saccharification that depends on the substrate (Yang B. et al., 2011). To overcome these limitations, several genetic modifications have been made in the past few years. The progress achieved in recent years for enhancing cellulase activity, pH and thermal stability are presented in Table 2.

**TABLE 1:** Industrial applications of cellulases.

Industry	Function	References
Biofuel and Biorefinery	• Enzymatic saccharification of lignocellulosic materials for the production of bioethanol, biobutanol, other solvents, organic acids, single cell protein, and lipids	Chandel et al., 2011; Gong et al., 2014; Annamalai et al., 2016

Paper and Pulp	• Improves quality of paper	Pere et al., 2001;
	• Deinking of paper wastes	Dienes et al., 2004
	• Biomechanical pulping using cellulases	
	• Biomodification of fiber properties	
	• Enhances the bleachability of softwood kraft pulp	

Textile	• Finishing of cellulose-based textiles	Belghith et al., 2001;
	• Stonewashing of jeans	Ibrahim et al., 2011
	• Biopolishing of cotton and other cellulosic fabrics	

Wine and Brewery	• Important role in fermentation processes	Gil and Vallès, 2001;
	• Improves the wine quality and stability by improving color extraction, skin maceration, must clarification, filtration	Claus and Mojsov, 2018
	• Improves the aroma of wines	
	• Reduces the viscosity of wort and improves the filterability	

Food Processing	• Macerating enzymes complex Increase the yield of juices	De Carvalho et al., 2008;
	• Improves texture and decrease viscosity of the nectars and purees from tropical fruits such as mango, peach, papaya, plum, apricot, and pear	Natarajan et al., 2018
	• Improves texture, flavor, and aroma properties of fruits and vegetables	

Agriculture	• Plant protoplast production, which can be also used to produce hybrid strains with desirable properties	Phitsuwan et al., 2012
	• Enhances growth of crops	
	• Capable of degrading the cell wall of plant pathogens in controlling the plant disease	
	• Enhances seed germination, plant growth and flowering, improves root system and increased crop yields	
	• Improves the soil quality and fertility	

Animal feed	• Improves feed value and performance of animals	Murad and Azzaz, 2010
	• Improves nutritional value of agricultural silage and grain feed	
	• Improves the quality of pork meat	
	• Reduces viscosity of high fiber rye- and barley-based feeds in poultry and pig	

Waste Management	• Cellulose containing wastes utilized to produce valuable products such as biofuels, chemicals, enzymes, sugars, energy sources for fermentation and improved animal feeds	Khan et al., 2016

Detergent	• Improves color brightness and removes dirt from the cotton blend garments	Olsen and Falholt, 1998
	• Removes rough protuberances for a smoother, glossier, and brighter-colored fabric	

**TABLE 2:** Recent progress in engineering cellulases.

Enzyme/Gene	Organism source	Engineering strategy	Improved characteristics	References
CMC-1	*Bos frontalis* metagenome	ep-PCR#	**Activity:** ↑ 2 fold	Wu et al., 2020
CBH	*S. cerevisiae*	CRISPR-Cas9	**Activity:** ↑ 85.9%	Li et al., 2020b
BaGH5	*Bacillus amyloliquefaciens*	Directed evolution	**Activity:** ↑ 1.6–4.1-fold **pH:** ↑15% at pH 4	Singh et al., 2020
BglA	*Clostridium thermocellum*	Directed evolution	**Thermostability:** ↑T_i_ by 6.4°C.	Yoav et al., 2019
EGLII	*Penicillium verruculosum*	Rational design	**Activity:** ↑ specific activity 15–21% **Thermostability:** ↑∼20% at 70°C	Bashirova et al., 2019
Cel5	*Gloeophyllum trabeum*	SDM^#^	**Activity:** ↑ K_*cat*_/K_M_ by 45 and 52% **pH:** ↑∼75%at pH 2	Zheng et al., 2018
GH12	*Streptomyces* sp.	ep-PCR	**Activity:** ↑∼1.7 fold **Thermostability:** ↑∼2 fold at 60°C **pH:** ↑∼2 fold at pH 5 and ∼2.6 fold at pH 6	Cecchini et al., 2018
Cel5A	*Bacillus agaradherans*	SDM	**Activity:** ↑∼1.5-3.4-fold	Saavedra et al., 2018
Cel5A	*Trichoderma reesei*	SDM	**Activity:** ↑ k_*cat*_/K_M_-1.3-1.8- fold **Thermostability:** ↑ T_1__/__2_-2.4-fold (80°C), 2.01-fold (70°C), 1.8-fold (60°C)	Akbarzadeh et al., 2018
GH5 Cel5E	*Clostridium thermocellum*	Rational design	**Activity:** ↑ 1.2–1.9 fold (CMC^#)^ ↑ 1.2–1.4 fold (barley β-glucan) **pH:** ↑∼1.5–2 fold at pH 6–8	Torktaz et al., 2018
Bgl Ks5A7	Kusaya gravy metagenome	Directed evolution	**Activity:** ↑ specific activity ∼1.5 fold **Thermostability:** ↑ from 45 to 65°C **pH:** ↑ 30% at pH 4.5	Cao et al., 2018
CtCel6	*Chaetomium thermophilum*	SDM	**Activity:**↑ 1.82 fold (β-D-glucan), 1.65 fold (PASC)and 1.43-fold (CMC) **Thermostability:** ↑ T_1__/__2_-1.4–2.4-fold	Han et al., 2018
CelA	*Caldicellulosiruptor bescii*	Rational design	**Activity:** ↑ 82 and 30%	Kim et al., 2017
Cel7A	*Hypocrea jecorina*	Directed evolution	**Activity:** ↑∼2-fold (PASC^#^) **Thermostability:** ↑Tm by 10.4°C ↑T_1__/__2_ by 44-fold	Goedegebuur et al., 2017
BglT	*Bacillus terquilensis*	Rational design	**Activity:** ↑ 64.4% AZO- barley β-glucan, ↑ k_*cat*_/K_M_∼1.5-fold **Thermostability: ↑** T_1__/__2_ by 3.86-fold (60°C), and 7.13-fold (70°C), ↑ Tm by 13.8°C	Niu et al., 2017
MeBglD2	Soil metagenome	SDM	**Thermostability:** ↑ optimal temp. by ∼10°C, ↑ Tm by 7°C and 9°C	Matsuzawa et al., 2017
Bgl	*Penicillium oxalicum*	Rational design	**Activity:** ↑ 65-fold	Yao et al., 2016
Bgl1	*Aspergillus niger*	ep-PCR	**Activity:** ↑ 156%	Larue et al., 2016
Bgl3A	*Talaromyces leycettanus*	SDM	**pH:** stability at broad range (pH 3-11)	Xia et al., 2016
Cel7B	*Trichoderma reesei*	Structure-guided evolution	**Activity: ↑** 4-fold (CMC) ↑∼1.4–2-fold (Avicel), ↑1.6–3-fold (MUC^#^) **Thermostability:** ↑Tm in 3°C ↑ T_1__/__2_ by ∼2-fold at 60 °C	Chokhawala et al., 2015

***#ep-PCR,** Error Prone PCR; **SDM,** Site Directed Mutagenesis**; CMC,** Carboxy Methyl Cellulose**; PASC,** Phosphoric Acid-Swollen Cellulose**; MUC,** 4-Methylumbelliferyl β-D-cellobioside.*

### Rational Design

This strategy involves the comprehensive knowledge of the protein structure. The process of rational design involves three steps (1) selection of appropriate enzyme, (2) identification of the amino acid sites to be altered, usually based on a high resolution crystallographic studies, and (3) characterization of the mutants (Prajapati et al., 2018). Site Directed Mutagenesis (SDM) allows modifications of amino acid sequences. The important residues near catalytic/substrate binding sites, which confer important properties, are analyzed. In an investigation, the substitution of non-aromatic residue at site 245 of endoglucanase Cel5A from *Acidothermus cellulolyticus* resulted in a 20% improvement in the enzyme activity (Baker et al., 2005). SDM of two amino acid residues (L167W or P172L) at the active site of β-glucosidase (Cel1A) from *T. reesei* led to enhancement in glucose sensitivity, pH and thermal stabilities. The Cel1A mutant displayed high glucose tolerance (50% of inhibition at 650 mM), enhanced thermostability (up to 50°C) and pH stability (4.5–9.0), in comparison with 40°C and pH 6.5–7.0 for the wild-type Cel1A (Guo et al., 2016).

Attempts have also been made to increase protein secretion in yeasts (Ashe and Bill, 2011; Traxlmayr and Obinger, 2012; Huang et al., 2017). Yeasts have the ability to express and secrete heterologous proteins and are widely used for production of recombinant proteins because of its additional ability for post-translational processing and high density fermentation (Damasceno et al., 2011). However, proteins are secreted in low titer as compared to the filamentous fungi (Zhang and An, 2010). Several strategies of engineering molecular chaperones and foldases, leader peptide sequence, optimization of the gene copy number, manipulation of promoter strength and codon optimization have been investigated.

#### Overexpression by Engineering Molecular Chaperones

The accumulation of various misfolded or unfolded proteins impairs the heterologous protein expression. Therefore, overexpression and engineering chaperones are known to cause correct folding and enhanced protein secretion that results in enhanced enzyme activity (Xiao et al., 2014). The overexpression of endoplasmic reticulum chaperone protein disulfide isomerase (Pdi1p) resulted in 53% enhanced specific bgl activity in recombinant *Saccharomyces cerevisiae* strains as compared to the wild-type (Tang et al., 2015). In another study, Li et al. (2018) overexpressed HAC1 (key transcription factor that regulates the unfolded protein response) that enhanced folding and secretion of the recombinant egl1 in *Pichia pastoris.* This resulted in 619% higher enzyme activity (91 U/ml) than that recorded in the wild-type strain. Yang F. et al. (2015) cloned β-glucosidase (*bgl*) from *Thermoanaerobacterium aotearoense* together with a chaperone (groES-groEL) in pGro7 in *E. coli.* The recombinants exhibited 9.2-fold higher specific enzyme activity than the strains lacking chaperones.

#### Engineering Leader Peptide Sequence

The secretion of proteins is majorly dependent on amino acid sequences and the structure of the protein. To maximize the secretion of a target protein, an appropriate combination of a leader peptide and a target protein is required (Oman and van der Donk, 2010). The leader peptide of the α-mating pheromone (*MF*α*1*SP) of *S. cerevisiae* is the most commonly used signal peptide (SP) for secretory production of heterologous proteins in *P. pastoris* (Brake et al., 1984). Zhu et al. (2010) observed enhanced endoglucanase activity (61.5%) by replacing native secretion signal sequence of the cellulase endoglucanase I (*eg1*) gene by MFα (α-mating pheromone) of *S. cerevisiae.* The *MF*α*1*SP directs the secretion of a variety of heterologous proteins in a numerous hosts, including *S. cerevisiae*, *Schizosaccharomyces pombe*, *P. pastoris* and mammalian cells (Brake et al., 1984; Oka et al., 1999; Lee et al., 2002; Kjaerulff and Jensen, 2005). Inokuma et al. (2016) constructed a gene cassette for cell surface display of β-glucosidase (bgl1) from *Aspergillus aculeatus* and endoglucanase II (eglII) from *T. reesei.* The SP sequences of gene cassettes were derived from *S. cerevisiae* SED1 (*SED1*SP), *Rhizopus oryzae* glucoamylase (*GLUA*SP), and *S. cerevisiae* α-mating pheromone (*MF*α1SP). The recombinant strains with the *SED1*SP displayed 1.3- and 1.9-fold higher bgl activity than the GLUASP and MFα1SP strains, respectively. There was no significant difference in extracellular egl activity of recombinant strains with the *SED1*SP and *MF*α1SP. The SP is effective in cell-surface display and secretory production of heterologous cellulolytic enzymes in various hosts.

Engineering of secretion signal peptides led to increased protein secretion in some cases. However, these synthetic secretory peptides are not beneficial for all heterologous proteins (Bae et al., 2015). Thus, it is necessary to identify target protein-specific secretion fusion partner. Therefore, the screening of a protein-specific translational fusion partners (TFP) from yeast genome-wide secretion leader library is a novel protein secretion system developed for the poorly secreted proteins in yeast (Bae et al., 2015). Following this technique various cellulases from different sources were investigated by screening an optimal translational fusion partner (TFP) as both secretion signal and fusion partner (Lee et al., 2017). Protein secretion and enzymatic hydrolytic activity of *Chrysosporium lucknowense* cbh2 were 2.4- and 1.4-fold higher than the native signal peptide and MFα, respectively. The enzyme activity of *Saccharomycopsis fibuligera* bgl1 was also 4.3- and 39.9-fold higher than the protein secreted by the native signal peptide and MFα, respectively. The function of target protein-specific TFP is still unknown, although it appears to play a vital role in correct folding of target proteins in the endoplasmic reticulum (ER) or trafficking to the golgi complex.

#### Optimization of Gene Copy Number

A genetically engineered *Aspergillus oryzae* was developed that simultaneously produced cbh, egl, and bgl by integrating multiple copies of the genes into the chromosomes. The recombinants possessed 5–16 copies of each of the cellulase genes, and thus, exhibited 10-fold enhancement as compared to those with single integration (Wakai et al., 2019). Li et al. (2018) observed 262 and 151% higher straw mushroom egl activity in recombinants with four and eight copies of gene than the host harboring single and four copies, respectively. Conversely, in some studies high copy number transformants did not produce high titer of recombinant proteins. In an investigation using a transformant with one copy of *Neosartorya fischeri* bgl3A gene displayed higher β-glucosidase activity than that with four copies of the gene (Xue et al., 2016). The reason for this could be loci integration. Therefore, it has been suggested that the integration locus, apart from gene copy numbers, might also affect protein expression.

#### Manipulation of Promoter Strength

Heterologous protein production depends on the availability of efficient promoter systems that allow a controlled and a high level of gene expression (Fitz et al., 2018). Manipulation of promoter strength has, therefore, been found successful in increasing gene expression. Zou et al. (2012) modified *cbh1* promoter of *T. reesei* by replacing the CREI binding sites (responsible for reducing promoter strength) with the binding sites of transcription activator ACEII and the HAP2/3/5 complex (positive regulator). The addition of a rigid α-helix linker in hybrid gene of cbh1 from *T. reesei* and e1 (endoglucanase) from *Acidothermus cellulolyticus* improved the efficiency of heterologous expression. This recombinant enzyme displayed enhanced thermostability as well as 39 and 30% enhanced FPase and CMCase activities, respectively. The effect of *cis* element on promoter strength had also been investigated by Kiesenhofer et al. (2017). The cellobiohydrolase (*cbh1*) and xylanase (*xyn1*) of *T. reesei* were transcriptionally activated by the regulatory protein Xyr1. Xyr1-binding sites (XBS) could be arranged in tandem or in inverted repeats. The inverted repeat (*cis* element) is known to play a vital role in inducing *xyn1* and *cbh1* promoters that allows enzyme induction (Kiesenhofer et al., 2017).

#### Codon Optimization

Codon optimization is a strategy for improving protein expression level in an organism by increasing translational efficiency of the target genes (Quax et al., 2015). Preferential usage of particular codons varies among different organisms. Replacing the rarely occurring codons with the preferred codons of the host expression system has been found successful in various attempts to strengthen the heterologous expression (Quax et al., 2015). The heterologous expression of codon optimized Cel6A of *T. reesei* was 10-fold higher than the wild-type gene. The secretory capacity of the recombinant Cel6A was improved further in high cell density fed-batch cultivation in comparison with that in shake flasks, which led to enhanced cell biomass and approximately 3.7-fold higher specific CMCase activity of the recombinants (Sun et al., 2016). Codon optimized egl1 of *T. reesei* was expressed at a higher level in *P. pastoris.* The enzyme activity increased to 1.24-fold (Akcapinar et al., 2011). The cellobiohydrolase activity of the synthetic *cbh2* gene from *T. reesei* was 2.02-fold higher than the native *cbh2* gene (Fang and Xia, 2015). Phadtare et al. (2017) cloned codon optimized endoglucanase gene of *Myceliophthora thermophila* (*Mt-egl*) and constitutively expressed in *P. pastoris*. Using recombinant Mt-egl, the sugar yields attained by hydrolyzing wheat bran and corn cobs were 421 and 382 mg/g, respectively. Pei et al. (2012) performed SDM to replace the rare codons for the N-terminal amino acid residues of *Thermoanaerobacterium thermosaccharolyticum bgl* in order to optimize *bgl* codons for expression in *E. coli*. The expression level of the recombinant improved from 6.6 to 11.2 U/mg. It also exhibited high tolerance to glucose and cellobiose. These studies have made amply clear that codon optimization is an effective strategy to express genes in suitable heterologous hosts.

### Directed Evolution

Directed evolution does not rely on a detailed structural and functional knowledge of proteins like rational design, rather it relies on the simple mechanism of random mutations and selection (Cobb et al., 2013). As shown in Figure 1, the target gene is first selected and then through random mutagenesis or gene recombination, a library of mutant genes is created. The resulting mutants are further screened for improved characteristics (Nannemann et al., 2011). This method has become a valuable tool for ameliorating various enzymes such as xylanases (Xu et al., 2016; Acevedo et al., 2017), laccases (Mateljak et al., 2019), phytases (Shivange et al., 2016; Körfer et al., 2018), amylases (Wu et al., 2018; Huang et al., 2019) and cellulases (Liu et al., 2014; Larue et al., 2016; Lin et al., 2016; Goedegebuur et al., 2017; Yang et al., 2017; Cao et al., 2018). Cao et al. (2018) modified β-glucosidase (Ks5A7) for improved thermostability, catalytic efficiency and resistance to glucose inhibition. Four rounds of random mutagenesis using error-prone PCR led to the substitution of five amino acids. The mutant displayed 1.5-fold enhanced specific activity than the wild-type enzyme and high glucose-tolerance with an *IC*_50_ of 1.5 M. The supplementation of mutant enzyme with Celluclast improved the glucose yield by 44% following hydrolysis. Cellobiohydrolase I (Cel7A) from *Hypocrea jecorina* was mutated through combinatorial approach. The variant comprised 18 mutated sites with a half-life 44 times higher than the wild-type enzyme, and 10.4°C enhancement in its thermal denaturation melting point (Goedegebuur et al., 2017). The variant of *A. niger* bgl obtained through this approach exhibited Tyr → Cys substitution at 305 position which intensely diminished transglycosylation activity that led to the inhibition of the hydrolytic reaction at high substrate concentrations (Larue et al., 2016). Error prone PCR technique has also been used extensively to produce proteins with new or improved characteristics. Using error prone PCR for exoglucanase (*cbhA*) of *Cellulomonas fimi*, three variants with improved enzymatic activity were selected out of 4000 clones. The specific enzyme activities of three variants were 1. 4-, 1. 3-, and 1.6-fold higher than the wild-type (Liu et al., 2014). *B. subtilis* endo-β-1,4-glucanase gene (*beg*) was mutated by error-prone PCR and DNA shuffling. Mutation resulted in the substitution at seven amino acid residues (K45E, I102Y, M112V, D226Y, D295E, L423S, and D460G). The thermostability of the variants was enhanced by 1.5-fold (Yang et al., 2017). Substitution of amino acid residues K353E and G117S of endoglucanase (Cel8M) from *E. coli* was observed to directly affect the substrate binding affinity that improved the enzyme activities by 1.4- and 1.6- fold, respectively (Lin et al., 2016).

The limitation with directed evolution technology is its requirement for a sensitive and efficient method for high throughput screening of a large number of mutants (Xiao et al., 2015). The development of novel high-throughput screening processes and strategies will extend further the application of directed evolution for various industrial enzymes. A few attempts have been made to combine both rational design and directed evolution to improve the properties of the enzymes (Chica et al., 2005; Lutz, 2010). The combined approach is also proving to be a successful route in protein engineering.

### Genetic Engineering Through Structural Modification

#### Glycosylation

Cellulases are glycoside hydrolases and genes encoding them are found in bacterial as well as fungal genomes (Dadwal et al., 2019). The fungal cellulases are both *N*- and *O*-glycosylated in their native form (Greene et al., 2015). Although the roles of glycosylation in enzyme function and structure have not been fully understood, glycosylation is known to modify various properties of cellulases including enhanced activity, thermostability, proteolytic and structural stability (Chung et al., 2019). Payne et al. (2013) demonstrated that *O*-glycosylated cellulase linkers play a role in cellulose-binding affinity, which suggests that the *O*-glycansmay be critical in cellulose binding. The addition of *O*-glycans led to enhanced proteolytic and thermal stability and cellulose-binding affinity of family 1 CBM (Taylor et al., 2012; Chen et al., 2014; Guan et al., 2015). The addition of an *N*-glycan on *Penicillium funiculosum* Cel7A at asparagine-194 via mutation of alanine-196 to serine led to 85% improvement in enzyme activity (Adney et al., 2009). It has been reported by Gupta et al. (2011) that deglycosylation of *T. reesei* Cel7A led to reduction in enzyme stability. Amore et al. (2017) reported that *N*-glycan mutants protect GH7 cbh of *T. reesei* (TrCel7A) against proteolysis. The mutants developed by removing the potential O-glycosylation sites displayed enhanced pH stability over a broader pH range (3.0–10.0). The saccharification efficiency of the mutant was also improved approximately equivalent to commercial β-glucosidase (Novozyme 188). The effect of four N-glycosylation sites (N224, NN295, N363, and N429) on bgl from *Aspergillus terreus* had been studied by Wei et al. (2013). The mutant obtained with no *N*-glycosylation displayed reduced activity and thermostability. The extracellular pNPGase activity of the transformant reached a level comparable with the commercial cellulase complex Accellerase 1000 (450 IU/mL). The *N*-glycosylation site N224 was also observed to play an important role in facilitating the proper folding of bgl. These investigations thus prove that the engineering and design of glycans is another approach to enhance cellulase activity and thermostability. Glycosylation is also known to modulate binding to cellulose and lignin (Chung et al., 2019). Overall, there is a need for in-depth understanding of multi-domain architectures of cellulases as there are variations in findings on the role of glycosylation in cellulases.

#### Computational Approach

Computational protein design offers an efficient method to evolve improved enzymes. The molecular dynamics simulations allow the identification of specific mutations that are beneficial for enzyme stability (Childers and Daggett, 2017). The substitution of extra glycine residue in cellulase from *Thermobifida fusca* (Cel9A-68) was identified to possess enhanced interdomain motions with increased overall flexibility as compared to the WT enzyme; this was observed as a rigid system. The presence of more flexible linkers resulted in higher cellulolytic activity. Thus, the linker mutations may prove to be an efficient way to improve enzyme activity (Costa et al., 2018).

The molecular dynamics (MD) simulations have been studied widely to modulate the thermostability of cellulases (Akcapinar et al., 2015; Jiang et al., 2016) and product binding sites of cellulases (Silveira and Skaf, 2014). The N-terminal region is responsible for enzyme thermostability in GH12 and could be a potential target for enzyme engineering (Jiang et al., 2016), while Akcapinar et al. (2015) used the MD simulations to validate the *in vitro* experiments. The Q126F, K272F and Q274V mutations were shown to exhibit stabilizing effect and higher thermostability compared to the native EGI of *T. reesei*. It was assumed that the increased thermostability in the mutants could be due to the changes in the distances of the catalytic cluster residues. The putative targets aimed at reducing the product inhibition have been investigated by Silveira and Skaf (2014) through analyzing the mutants by MD simulation. The D262A, Y381A and W376A mutations in TrCel7A were identified for product inhibition.

The computational design has also been utilized to negatively supercharge the surface of cellulase enzyme (Whitehead et al., 2017), as it has been known that negatively supercharging cellulases could reduce inhibition by lignin. Therefore, following this strategy the cellulase can be made highly resilient to impede its inactivation by lignin. The *in silico* engineering in disulfide bond of eglII from *Penicillium verruculosum* led to improved variants. Improvement in specific enzyme activity was 15–21%. The engineered variant resulted in a rigid globular structure leading to enhanced thermostability (Bashirova et al., 2019). Liu et al. (2010) used MD simulations to understand the role of N-terminus Ig-like domain of Cel9A from *Alicyclobacillus acidocaldarius.* This domain was found to stabilize the catalytic domain that helps in keeping it folded in the favorable conformation. These findings may enable development of more robust and stable cellulases. Sprenger et al. (2016) utilized MD simulation in studying the stability of bacterial LPMOs in ionic liquids (ILs). Increasing the rigidity of loops and engineering residues near the active site were observed to further increase stability in ILs. This strategy provides a step toward engineering LPMOs to function efficiently in enzyme cocktails.

## Genome Engineering by CRISPR/Cas 9 System

CRISPR/Cas9 (clustered regularly interspaced short palindromic repeats/CRISPR-associated protein 9) is an emerging genome editing technology which has been used in various organisms including some filamentous fungi (Nødvig et al., 2015). This system consists of two components, a Cas9 endonuclease and a single chimeric guide RNA (sgRNA). The sgRNA is 20 bp protospacer sequence which binds to the target site. The mature gRNA guides Cas9 to the target site and Cas9 then introduces a double-strand break (DSB) in the target DNA. The DSB can then be repaired by the host cell repair systems. The mechanism allows random insertions and deletions within the target sequence, therefore this system can be used both for the insertion and deletion of genes (Zhao et al., 2018). Using this technology, Liu et al. (2017) generated *Myceliophthora thermophila* and *Myceliophthora heterothallica* strains with significantly enhanced cellulase production. Multigene disruption of the genes involved in cellulase production (*cre-1*, *res-1*, *gh1-1*, and *alp-1*) led to hypercellulase production up to 5–13 fold. Even the deletion of single gene *cre-1* from *M. thermophila* (Δ*Mtcre-1*) resulted in 3.7-fold higher protein secretion in avicel medium. Similarly, *M. heterothallica* mutant (Δ*Mhcre-1*) exhibited a marked improvement in cellulase activity and secreted protein levels. An attempt to engineer *S. cerevisiae* following this technique was made by integrating *sestc* expression cassettes containing glyceraldehyde-3-phosphate-dehydrogenase gene (gpd) promoter of *Agaricus bisporus* (Yang P. et al., 2018). The endo-1,4-β-glucanase and exo-1,4-β-glucanase activities of the recombinants were 35.3-and 23-fold higher than the wild-type *S. cerevisiae*. The replacement of endogenous promoter of *clr-2* (CLR-2 is the transcription factor that regulates the expression of cellulases) with the β*-tubulin* promoter in *Neurospora crassa* led to enhancement in mRNA cellulase expression (Matsu-ura et al., 2015). The mRNA expression levels of *cbh-1*, gh5-1 (egl), and gh6-2 (cbh) increased to 68.3 ± 25. 5-, 1724.3 ± 538.1- and 14.6 ± 5.3-fold, respectively.

The introduction of CRISPR/Cas9 genome-editing technology has triggered a revolution in genetic engineering due to its versatility, high efficiency and easy operation. The application of this technology for hypercellulase production is still in its infancy. Further development and understanding of this system could provide a new opportunity for improving the production of cellulolytic enzymes.

**FIGURE 4:**
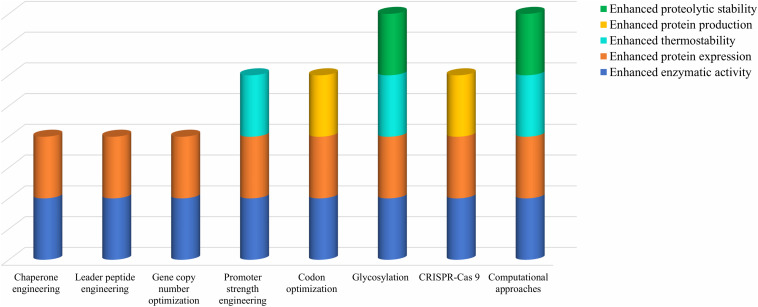
Comparative analysis of various genetic and metabolic engineering strategies in ameliorating cellulase characteristics.

Various engineering strategies have been attempted in order to improve the cellulase characteristics but no single technique is able to provide all the beneficial characteristics altogether. Figure 4 depicts the comparative analysis of several engineering strategies discussed above in ameliorating cellulase characteristics.

## Engineering for Glucose Tolerant β-Glucosidase

β-Glucosidase (bgl) is a key enzyme that catalyzes the hydrolysis of the β-1,4 linkages of cellobiose to liberate glucose. The hydrolysis step is usually known to be a limiting step in the conversion of lignocellulosics to sugars because of the accumulation of glucose as the reaction product (Kim, 2018). The presence of glucose interferes with the binding of the substrate to the active site that reduces the reaction rate (Singhania et al., 2013). Many bgls are known to be inhibited by glucose that results in reduced catalytic activity (Leite et al., 2008). This may limit the industrial applications of these enzymes, therefore, there is a search for bgls with high enzymatic and catalytic activity even at high glucose concentrations. Many genetic engineering approaches have been attempted in order to evolve improved bgls. A novel bgl (bgl6) isolated from a metagenomic library, engineered by the substitutions of three amino acids led to glucose tolerance with IC_50_ of 3.0 M. supplementation of the mutant bgl to Celluclast 1.5 L significantly improved the glucose yield by 30% from the pretreated sugarcane bagasse (SCB) (Cao et al., 2018). The substitutions of two amino acids L167W and P172L in GH1 β-glucosidase of *Trichoderma harzianum* (Thbgl) and Cel1A (bgl II) of *T. reesei* were identified as important amino acid residues involved in glucose tolerance (Guo et al., 2016; Santos et al., 2019). Mutant (Thbgl-Mut) showed remarkable improvement in glucose release during the saccharification reaction in different lignocellulosic substrates. A twofold enhancement in the production of bioethanol was also achieved using Thbgl-Mut. The mutant displayed enhanced thermostability by 10°C in comparison with the wild-type enzyme (Santos et al., 2019). Cel1A mutant of *T. reesei* also displayed enhanced pH stability and thermostability as well as 2.4-fold enhanced specific activity as compared to the wild-type Cel1A (Guo et al., 2016). Two mutations D237V and N235S in bgl from *Humicola insolens* (bglhi) led to decline in tolerance to monosaccharides (Meleiro et al., 2017). Following semi-rational engineering approach, Fang et al. (2016) obtained bgl1A (bgl isolated from a marine microbial metagenomic library) variant A24S/F297Y with improved ethanol tolerance. The variant contained additional hydrogen bonds which further improved thermostability and pH stability. Random mutagenesis in Td2F2 (a metagenomic bgl) resulted in a mutation at Asn223 residue located between subsites +1 and +2. The variant displayed reduced glucose tolerance, transglycosylation activity as well as drastically changed substrate specificity. This study concluded that structure between subsites +1 and +2 might be important for the glucose tolerance and substrate specificity of Td2F2 (Matsuzawa et al., 2016). The mutant β-glucosidase from *Agrobacterium tumefaciens*, with increased hydrophobicity at +1 subsite and hydrophobicity and steric at +2 subsite, displayed 2.2-fold higher tolerance to glucose (Goswami et al., 2017).

The engineering methods have permitted new insights into the structure/function relationship of the enzymes, which may prove beneficial in understanding the regulation and catalytic properties of bgls. Better understanding of regulatory mechanisms for glucose tolerance and stimulation will aid in evolving robust enzymes that can be used in a wide range of industrial applications. Besides engineering the enzymes for ameliorating the characteristics, modification of bacterial and fungal host cells for improving the production of bgls is also desirable (Singhania et al., 2017).

Although several attempts have been made in evolving ameliorated cellulases by various protein/genetic engineering strategies, none of these has been used on a large scale in saccharifying lignocellulosics for producing bioethanol. Noteworthy developments in this domain are expected in the near future.

## Microbial Strain Engineering for Consolidated Bioprocessing

Due to high costs involved in cellulose to sugar conversion process and inefficient fermentation of both hexose and pentose sugars, the commercial bioethanol has become economically unviable. Consolidated bioprocessing (CBP) is an attractive approach which offers more efficient conversion of lignocellulosics to ethanol, since the process combines cellulase production, cellulose hydrolysis and ethanol fermentation in a single step (Liu et al., 2016). Over the past few years, many attempts have been made to engineer strains (fungal, bacterial as well as yeast) for its application in CBP (Den Haan et al., 2015). Recently, a thermophilic fungus *Myceliophthora thermophila* was engineered to produce ethanol from glucose and cellobiose. Metabolic engineering by overexpressing a glucose transporter GLT-1 or the cellodextrin transport system (CDT-1/CDT-2) from *N. crassa* led to 131 and 200% enhanced ethanol production from glucose and cellobiose, respectively as compared to the wild-type (Li et al., 2020a). In another study, a non-cellulolytic yeast *Yarrowia lipolytica* was made cellulolytic for its use in consolidated bioprocessing of cellulose. An engineered *Y. lipolytica* was developed by coexpression of six cellulolytic enzyme components (β-glucosidases, the CBH II, and EG I and II from *T. reesei*, and the CBH I from *N. crassa*). The study revealed viability of *Yarrowia* strains in CBP for the production of biofuel and other chemical precursors (Guo et al., 2017). *Caldicellulosiruptor besci* is a strain which does not produce ethanol. A ldh mutant of the strain with incorporation of NADH-dependent AdhE from *C. thermocellum* led to production of ethanol at 75°C. Although, the yield and ethanol titer were low, the study has shown the possibility of improving the bacterial strain (Chung et al., 2015). Despite several challenges, further investigations are called for developing CBP-organisms in order to saccharify cellulosic biomass and bioethanol production with improvement in the carbohydrate metabolizing enzymes and fermentation abilities of various microorganisms.

## Multifunctional Cellulases and Cellulosomes

Microbial cellulase systems in the natural form exist as free enzymes, multifunctional enzymes or multi-enzyme complexes (cellulosomes), for enzymatic degradation of plant cell walls (Himmel et al., 2010). Multifunctional enzymes possesses two or more catalytic modules and/or Carbohydrate Binding Modules (CBMs) important for the degradation of plant cell walls that led to improved concerted action on cellulosic substrates. Four different types of multifunctional enzymes are known till date: cellulase–cellulase, hemicellulase–cellulase, hemicellulase–hemicellulase, and hemicellulase–carbohydrate esterase systems (Himmel et al., 2010). Cellulosomes are multi-component complex made up of non-catalytic scaffoldins and comprising a CBM for substrate binding and multiple cohesin modules for integrating dockerin-bearing enzymatic subunits. With the advent of molecular biology and other synthetic biology techniques, multifunctional enzyme chimeras artificial cellulosomes are being made to recreate these natural systems for enhanced biomass degradation (Artzi et al., 2016).

Several attempts have been made to develop bifunctional cellulases. Two novel genes xylanase (XylC) and a cellulase (CelC) isolated from a camel rumen metagenome, had been used for constructing a bifunctional xylanase-cellulase chimera. The modular XylC is composed of a xylanase (Xyn) domain, a CBM, and a carbohydrate esterase (CE) domain. Three chimeras including Xyn-CBM-Cel, Cel-CBM, and Cel-CBM-CE were made by fusing the domains of XylC to CelC. The Cel-CBM-CE, and Xyn-CBM-Cel displayed a marked improvement in CMCase activities; 883 and 1979 U/mg in specific activity as compare to the wild-type CelC (688 U/mg). The CelC fusions displayed enhanced activity on rice and barley straws compared to the wild-type CelC (Ghadikolaei et al., 2017). The recombinant bifunctional endoglucanase-xylanase gene (*BhCell-Xyl*) of polyextremophilic bacterium *Bacillus halodurans* was cloned and expressed in *E. coli* (Rattu et al., 2016) as well as *Pichia pastoris* (Prabhu et al., 2016). The recombinant bifunctional enzyme expressed in *E. coli* displayed CMCase and xylanase activities optimally at pH 6.0 and 60°C. While the *P. pastoris* recombinant exhibited activity optimally at 60°C and pH 6.0–8.0.

Using synthetic biology approach, a GH5 endoglucanase and GH48 exoglucanase of *Thermomonospora fusca* was developed into a bifunctional enzyme (Morais et al., 2012). The concerted action of the two selected cellulases(Cel5A and Cel4bA) was superior to the bifunctional enzyme. A chimeric protein developed by the fusion of carbohydrate binding module (CBM) of CtLic26A-Cel5E (endoglucanase H or CelH) from *Clostridium thermocellum* to the C-terminus of DturCelA (thermostable endoglucanase) from *Dictyoglomus turgidum*. At acidic pH, chimeric DturCelA exhibited higher relative activity (20%) than the native form (0%). It also retained 100% enzymatic activity at 70°C (Cattaneo et al., 2018). The CBMs are known to bring the catalytic domains close to the target substrate, leading to enhancement in the rate of catalysis (Gilbert et al., 2013). The chimeras developed with CBM fusion displayed enhanced stability at extreme pH, higher activity and affinity. The fusion of CtGH5-F194A (mutant endoglucanase) with a β-1,4-glucosidase (CtGH1 from *C. thermocellum*) exhibited a 3–5 fold higher catalytic efficiency and improved thermostability for endoglucanase and β-glucosidase activities. Chimera showed 1.6-fold higher glucose yield from the pretreated sorghum stalks as compared to the mixed enzyme CtGH1 and CtGH5-F194A (Nath et al., 2019). In order to achieve enhanced cellulosic biomass degradation, engineering of multifunctional enzymes demand continuous research efforts and understanding of synergistic action of various enzymatic paradigms.

Noteworthy developments have been made on cellulosome production and application, although there is a gap in its industrial use. The development of artificial cellulosomes is based on the interaction between cohesin and dockerin modules. The designer cellulosomes allow the blend of enzymes from diverse organisms in a single scaffold. The chimeric scaffold consists of a CBM module which allows substrate targeting and several cohesin modules of divergent species with different specificities (Gonçalves et al., 2014). The designer cellulosomes allow to regulate the number, conformation and location of selected enzymes and their incorporation into a given chimeric scaffold (Kahn et al., 2019). Various strategies have been used in the development of multi-enzyme complexes, based on enzyme type, scaffold material, and conjugation technique (Gonçalves et al., 2014). Kahn et al. (2019) developed a cellulosome complex using enzymes from *C. bescii*. Various dockerin modules were grafted onto enzymes and inserted into a chimeric scaffold with their matching cohesins. The resultant cellulosome exhibited 1.7–1.8-fold higher enzyme activity than the native cellulosome of *C. thermocellum* at 75°C after 72 h (Kahn et al., 2019). The cellulosomes active on cellulose and hemicellulose with dockerin fused variant of laccase from the aerobic bacterium *Tthermobifida fusca* was designed. The complex produced twofold higher reducing sugar from wheat straw as compared to the same system devoid of laccase (Davidi et al., 2016). The incorporation of accessory enzymes such as LPMOs and expansins into designer cellulosomes has also been investigated and proved to be an efficient strategy in enhancing the yield of reducing sugars from biomass by glycoside hydrolases (Gefen et al., 2012; Artzi et al., 2016; Chen et al., 2016). The addition of thermostable scaffold into the designer cellulosomes (comprising endoglucanase Cel8A, exoglucanase Cel48S and β-glucosidase (bglA) mutants from *C. thermocellum*) displayed a 1.7-fold improvement in cellulose hydrolysis as compared to what achieved by cellulosomes containing wild-type enzymes. The chimeric scaffold consists of thermostable components derived from thermophilic microbes (Moraïs et al., 2016).

The structure and function of natural cellulosomes have not been adequately understood; the progress in the field of designer cellulosomes has broadened the scope on these aspects. Cellulosome has drawn attention because of its low cost and its high efficiency in deconstruction of lignocellulosics (Teeravivattanakit et al., 2016). The investigations have provided some insights on reconstitution of cellulosome complexes either *in vitro* or cell surface display (Hu and Zhu, 2019). The reconstitution of cellulosome complexes should focus on the characteristics of natural cellulosome, full-length cellulosome and complex multienzyme system. The recent developments in the cellulosome field would encourage further developments on the novel forms of artificial cellulosomes with maximal cellulolytic efficiency. Nanotechnology has also been applied recently for the synthesis of cellulosomes. The use of nanomaterials in immobilizing scaffolds allows high specific surface area and minimum diffusional limitation. The nanomaterials have been proved to be a potential scaffold for cellulase immobilization (Gunnoo et al., 2016; Hyeon et al., 2016; Lu Z. et al., 2019).

## Cellulases Retrieved From Metagenomes

Metagenomics has been proved to be useful in the discovery of novel cellulases through functional and sequence-based approaches (Popovic et al., 2015). Metagenomics allows retrieval of genes encoding biocatalysts from culturable as well as non-cultural microbes. Many cellulase genes have been identified from environmental metagenomes (Garg et al., 2016; Matsuzawa and Yaoi, 2016; Pandey et al., 2016; Zhou et al., 2016), hindgut contents of termites (Duan et al., 2017; Tsegaye et al., 2018), rumen (Ko et al., 2015; Song et al., 2016; Do et al., 2018), compost (Lemos et al., 2017; Lee et al., 2018), oil reservoirs (Lewin et al., 2017), and sludge from a biogas reactor (Wei et al., 2015). Very few of them have, however, been characterized adequately (Garg et al., 2016; Song et al., 2016; Lee et al., 2018).

Metagenomic cellulases have been subjected to protein engineering. Using directed evolution strategy, a metagenome-derived β-glucosidase of Bgl1Dwas engineered for improving the activity and thermostability (Yin et al., 2019). The mutant Bgl1D with five amino acid substitutions (S28T, Y37H, D44E, R91G, and L115N) displayed 23- and 10-fold enhanced catalytic efficiency (kcat/Km) and thermostability as compared to the wild-type Bgl1D. SDM revealed that Asp44 residue is crucial for enzymatic activity of Bgl1D (Yin et al., 2019). Directed evolution strategy following ep-PCR was employed to a ruminal cellulase gene (CMC-1) from a metagenomic library constructed from Yunnan gayal (*Bos frontalis*). A mutant EP-15was selected from a mutant library that showed twofold higher cellulase activity, enhanced pH and thermostability compared to the wild-type enzyme (CMC-1) (Wu et al., 2020).

## Conclusion

The requirement of improving characteristics of cellulases such as thermostability and high enzymatic activity for cellulose saccharification has led to a spurt of interest in genetic and protein engineering studies. The diverse array of intricacies in biological systems of various cellulolytic microbes may pose serious hurdles in evolving novel strategies for the production of robust cellulases. The strategies to evolve hypercellulase production enabled to understand the mechanism of cellulase hyperproduction, improve genetic engineering approaches along with metabolic engineering and systems biology, optimize parameters for enzyme induction and create suitable and useful gene assembly for cellulase production. Computational studies have also been useful in this regard because it provides insight into the information that that cannot be obtained through experimental study. Cellulase genes can also be retrieved from environmental metagenomes, which can be exploited for potentially desirable applications. Various strategies have been evolved to produce cellulases with improved efficiency and characteristics. The developments in genetic and metabolic engineering in addition to systems and synthetic biology would help in addressing the issue of developing novel strains and/or enzymes. The genetic engineering strategies are proving to be useful in transforming cellulase production. Further advancements in metabolic networks, gene assembly, optimization strategies for cellulase synthesis pathways, identification of novel multifunctional cellulases and cellulosomes can lead to exciting developments in enzyme production and their applications in industrial processes.

## Author Contributions

TS conceived the topic of the manuscript and synopsis, provided the critical feedback, and helped in shaping the manuscript. AD surveyed the literature and wrote the manuscript as per synopsis. SS provided the critical feedback and reviewed the final manuscript. All the authors have read and finalized the manuscript.

## Conflict of Interest

The authors declare that the research was conducted in the absence of any commercial or financial relationships that could be construed as a potential conflict of interest.

## Footnotes

1 www.cazy.org
